# BioNLP Shared Task - The Bacteria Track

**DOI:** 10.1186/1471-2105-13-S11-S3

**Published:** 2012-06-26

**Authors:** Robert Bossy, Julien Jourde, Alain-Pierre Manine, Philippe Veber, Erick Alphonse, Maarten van de Guchte, Philippe Bessières, Claire Nédellec

**Affiliations:** 1 Mathématique Informatique et Génome, Institut National de la Recherche Agronomique, INRA UR1077 - F78352 Jouy-en-Josas, France; 2 PredictiveDB - 16, rue Alexandre Parodi - F75010 Paris, France; 3 MICALIS, Institut National de la Recherche Agronomique, UMR1319 - F78352 Jouy-en-Josas, France

## Abstract

**Background:**

We present the BioNLP 2011 Shared Task Bacteria Track, the first Information Extraction challenge entirely dedicated to bacteria. It includes three tasks that cover different levels of biological knowledge. The Bacteria Gene Renaming supporting task is aimed at extracting gene renaming and gene name synonymy in PubMed abstracts. The Bacteria Gene Interaction is a gene/protein interaction extraction task from individual sentences. The interactions have been categorized into ten different sub-types, thus giving a detailed account of genetic regulations at the molecular level. Finally, the Bacteria Biotopes task focuses on the localization and environment of bacteria mentioned in textbook articles.

We describe the process of creation for the three corpora, including document acquisition and manual annotation, as well as the metrics used to evaluate the participants' submissions.

**Results:**

Three teams submitted to the Bacteria Gene Renaming task; the best team achieved an F-score of 87%. For the Bacteria Gene Interaction task, the only participant's score had reached a global F-score of 77%, although the system efficiency varies significantly from one sub-type to another. Three teams submitted to the Bacteria Biotopes task with very different approaches; the best team achieved an F-score of 45%. However, the detailed study of the participating systems efficiency reveals the strengths and weaknesses of each participating system.

**Conclusions:**

The three tasks of the Bacteria Track offer participants a chance to address a wide range of issues in Information Extraction, including entity recognition, semantic typing and coreference resolution. We found commond trends in the most efficient systems: the systematic use of syntactic dependencies and machine learning. Nevertheless, the originality of the Bacteria Biotopes task encouraged the use of interesting novel methods and techniques, such as term compositionality, scopes wider than the sentence.

## Background

### Motivation and related work

The extraction of molecular events from the scientific literature is the most popular task in Information Extraction (IE) challenges applied to biology, such as in the LLL [1], BioCreative Protein-Protein Interaction Task [2], or BioNLP [3] challenges. Since the BioNLP 2009 shared task [4], this field has evolved from the extraction of a unique binary interaction relation between proteins and/or genes toward a broader acceptation of biological events, including localization and transformation [5].

The study of bacteria has numerous applications for health, food and industry, and overall, they are considered to be organisms of choice for the recent integrative approaches in systems biology because of their relative simplicity and the extent of the current knowledge. However, the current range of available vhallenges far from reflects the diversity of the potential applications of text mining to biology. The full understanding of a bacterial cell requires a wide range of levels of knowledge among which molecular mechanisms are only one aspect. Microbiologists also require information about the cell life cycle, cell structure, the detailed environment of the bacteria, and its phylogenetic position. The Bacteria Track of the BioNLP 2011 Shared Task gathers three Information Extraction tasks targeted at three different levels of knowledge on bacteria. Thus, we have the first set of IE challenges that are fully dedicated to bacteria and that encompass a wide range of knowledge levels.

At the nomenclatural level, the Bacteria Gene Renaming task challenges the participants to extract gene renaming acts and other gene synonymy mentions from the PubMed abstracts. At the molecular level, the Bacteria Gene Interaction is a more "classic" gene and protein interaction extraction task. Finally, we present the Bacteria Biotopes task, which aims at extracting information about bacteria habitats and biotopes as well as the places that they live.

### Bacteria Gene Renaming

Gene renaming is a frequent phenomenon, especially for model bacteria where there has been little to no effort toward the standardization of the nomenclature, and naming conventions are not strictly enforced. Moreover, the history of bacterial gene naming has led to drastic numbers of homonyms and synonyms. For example, many genes of *Bacillus subtilis *were renamed in the middle of the 1990s, so that the new names matched those of the *Escherichia coli *homologs.

**Figure 1:**
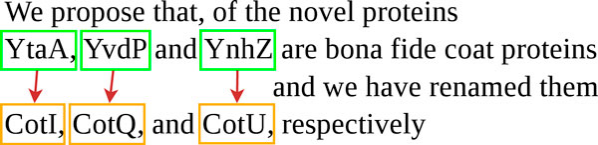
**Example of gene renaming relations**.

Hence, the abundance of gene synonyms that are not morphological variants is high compared to eukaryotes. Synonyms are often missing, or erroneous, in gene databases. Specifically, databases often omit old gene names that are no longer used in new publications but that are critical for an exhaustive bibliography search. Polysemy makes the situation even worse because old names frequently happen to be reused to denote different genes. A correct and complete gene synonym table is crucial to biology studies, for example, when integrating large-scale experimental data using distinct nomenclatures. Indeed, this information can save a substantial amount of bibliographic research time. The Rename task is a new task in text-mining for biology, that aims at extracting explicit mentions of renaming relations. The motivation of the Rename task is to keep bacteria gene synonym tables up to date. Additionally, it is a critical step in gene name normalization that is needed for further extraction of biological events such as genic interactions. The goal of the Rename task is illustrated by Figure 1. It consists of predicting renaming relations between text-bound gene names that are given as input. The only type of event is *Renaming *, for which both arguments are of type *Gene*. The event is directed, and the former and the new names are distinguished. Genes and proteins were not distinguished because of the high frequency of metonymy in renaming events. In the example of Figure 1, "YtaA", "YvdP" and "YnzH" are the former names of three proteins that were renamed "CotI", "CotQ" and "CotU", respectively.

### Bacteria Gene Interactions

Gene and protein interactions are not formulated in the same way for eukaryotes and prokaryotes. Descriptions of interactions and regulations in bacteria include more knowledge about their molecular actors and mechanisms, compared to the literature on eukaryotes. Typically in the bacteria literature, the genic regulations are more likely expressed by the direct binding of the protein, while in the eukaryote literature, non-genic agents related to environmental conditions are much more frequent. The bacteria Gene Interaction task (GI) is based on [6], which is a semantic re-annotation of the LLL challenge corpus [1], for which the description of the GI events in a fine-grained representation includes the distinction between expression, transcription and other action events, as well as different transcription controls (e.g., regulon membership, promoter binding). The entities not only are protein agents and gene targets but also extend to families, complexes and DNA sites (binding sites, promoters) to better capture the complexity of the regulation at a molecular level. The task consists of relating the entities with the relevant relations.

**Figure 2:**
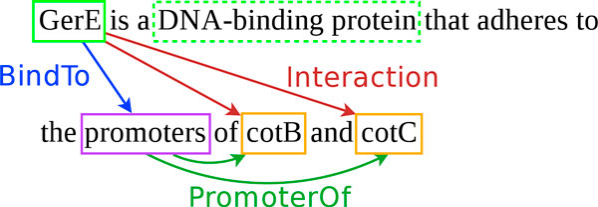
**Example of gene interaction relations**.

The goal of the GI task is illustrated by Figure 2. The genes "cotB" and "cotC" are related to their two promoters, which are not named here, by the relation *PromoterOf*. The protein "GerE" is related to these promoters by the relation "BindTo". As a consequence, "GerE" is related to "cotB" and "cotC" by an *Interaction *relation. According to [5], the need to define specialized relations replacing one unique and general interaction relation was raised in [7] for extracting genic interactions from text. An ontology describes the relations and entities [8], representing a model of gene transcription to which biologists implicitly refer in their publications. Therefore, the ontology is mainly oriented toward the description of a structural model of genes, with molecular mechanisms of their transcription and associated regulations.

### Bacteria Biotopes

The Bacteria Biotope (BB) task consists of extracting bacteria location events from Web pages, in other words, citations of places where a given species lives. It is the first step toward linking information on bacteria to ecological information at the molecular level.

According to NCBI statistics, there are nearly 900 bacteria with complete genomes, which account for more than 87% of the total complete genomes. Consequently, molecular studies in bacteriology are shifting from species-centered to full diversity investigations. The current trend in high-throughput experiments targets diversity-related fields, typically phylogeny or ecology. In this context, adaptation properties, biotopes and biotope properties become critical information. Illustrative questions in the field are as follows:

• Are some phylogenetic groups specialized to given biotopes?

• What are common metabolic pathways of species that live in given conditions, especially species that survive in extreme conditions?

• What are the molecular signaling patterns in host relationships or population relationships (*e.g*., in biofilms)?

Recent metagenomic experiments produce molecular data that are associated with a habitat rather than a single species. This scenario raises new challenges in computational biology and data integration, such as identifying known and new species that belong to a metagenome. Not only will these studies require comprehensive databases that associate bacterial species to their habitat but also they will require a formal description of the habitats for property inferences.

The bacteria biotope description is potentially very rich because any physical object, from a cell to a continent, can be a bacterial habitat. However, these relations are much simpler to model than with general formal spatial ontologies. A given place is a bacterial habitat if the bacteria and the habitat are physically in contact, while the relative position of the bacteria and its dissemination are not of specific interest. The information on bacterial habitats and properties of these habitats is very abundant in the literature, especially in the Systematics literature (*e.g*., *International Journal of Systematic and Evolutionary Microbiology*); however, it is rarely available in a structured way [9,10]. The NCBI GenBank http://www.ncbi.nlm.nih.gov/ nucleotide *isolation source *field and the JGI Genome OnLine Database ([11]) *isolation site *field are incomplete with respect to microbial diversity and are expressed in natural language. The two critical missing steps in terms of biotope knowledge modeling are (1) the automatic population of databases with organism/location pairs that are extracted from text, and (2) the normalization of the habitat name with respect to the biotope ontologies. The BB task aims mainly at solving the first information extraction issue. The second classification issue is handled through the categorization of locations into eight broad types.

From a linguistic point of view, the BB task differs from other IE molecular biology tasks while it raises some issues that are common to biomedicine and some of the more general IE tasks. The documents are scientific Web pages that are intended for non-experts such as encyclopedia notices. Documents are structured as encyclopedia pages, with the main focus on a single species or a few species of the same genus or family. The information is dense compared to scientific papers, and the frequency of anaphora and coreferences is unusually high. Location entities can be denoted by named entities, especially geographic locations and bacteria host species names. However, other locations are denoted as noun phrases or adjectives with no clear boundaries.

## Methods

### Bacteria Gene Renaming

#### Corpus annotation methodology

**Figure 3:**
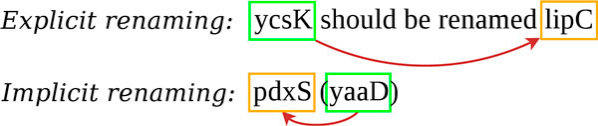
**Common types of renaming sentences**.

The Rename task corpus is a set of 1,644 PubMed references of bacterial genetic and genomic studies, including the title and abstract. Figure 3 presents the most common forms of renaming.

The main intent during the corpus creation process was the enrichment of mentions of gene renaming or gene synonymy; indeed, these mentions are extremely scarce. A first set of 23,000 documents was retrieved, identifying the presence of the bacterium *Bacillus subtilis *in the text and/or in the MeSH terms. *B. subtilis *documents are especially rich in renaming mentions.

As a second filtering step, we selected documents using two distinct criteria:

1. mentions of at least two gene synonyms, as recorded in the fusion of seven *B. subtilis *gene nomenclatures, leading to a set of 703 documents.

2. renaming expressions from a list that we manually designed and tested (*e.g*., "rename", "also known as"). Unexpectedly, these documents contained very few gene renamings, but instead contained renamings concerning other types of biological entities (*e.g*., protein domains, molecules, cellular ultrastructures). This criterion allowed us to add 941 documents.

Approximately 70% of the documents (1,146) were kept in the training data set. The remainder were split into the development and test sets, containing 246 and 252 documents, respectively. Table 1 gives the distribution of genes and renaming relations per corpus. Gene names were automatically annotated in the documents with the nomenclature of *B. subtilis*. Gene names involved in renaming acts were manually curated. Among the 21,878 genes mentioned in the three corpuses, 680 unique names are involved in renaming relations, which represent 891 occurrences of genes.

**Table 1:** Rename corpus size.

	***Training + Dev***.	*Test*
Documents	(1,146 + 246) 1,392	252 (15%)
Gene names	18,503	3,375 (15%)
Renamings	373	88 (24%)

The reference annotation of the Rename task corpus was performed in two steps, a first annotation step by Science and Technology Information professionals with initial specifications and a second verification step by bioinformaticians and computational linguists. The documents were annotated using the Cadixe editor http://caderige.imag.fr/Cadixe, following detailed annotation guidelines that were largely modified in the process. These adjustments were motivated by the manual analysis of observed discrepancies between annotators [12].

Given the number of ambiguous annotations, we designed a detailed typology to justify the acceptance or rejection decisions in seven different sub-cases.

The next three cases are not gene renamings (Table 2).

**Table 2:** Negative examples of the Rename task.

***Protein encoding***
*PMID 8969499: *The putative products of ORFs yeaB (Czd protein), yeaC (MoxR), yebA (CNG-channel and cGMP-channel proteins from eukaryotes),
***Genetic homology***
*PMID 10619015: *Dynamic movement of the ParA-like Soj protein of *B. subtilis *and its dual role in nucleoid organization and developmental regulation.
***Operon ***| ***Regulon ***| ***Family***
*PMID 3127379*: Three promoters direct transcription of the sigA (rpoD) operon in *Bacillus subtilis*.

Entity names of diverse types (gene, protein, operon) are underlined.

**Protein encoding **relation occurs between a gene and the protein it codes for. Some mentions may look like renaming relations. The example presents the gene "yeaC" coding for "MoxR". No member of the pair is expected to replace the other one.

**Homology **measures the similarity between gene or protein sequences. Most of the homology mentions involve genes or proteins from different species (orthologs). The others compare known gene or protein sequences of the same species (paralogs). This scenario could be misleading because the similarity mention could look like biological proof-based relations, as between "ParA" and "Soj" in Table 2.

**Operon, regulon or family **renaming involves objects that may look like genes, proteins or simple aggregations of gene or protein names but that are perceptibly different. The objects represent more than one gene or protein and the renaming does not necessarily affect all of them. More problematic, their name could be the same as one of the genes or proteins they contain, as in the example where "sigA" and "rpoD" are operons but are also known as gene names. Here, "sigA" (and thus "rpoD") represents at least two different genes. For the sake of clarity, operons, regulons and families are rejected, even if all of the genes are clearly named, as in an aggregation.

Three positive examples of renaming relations are shown in Table 3. Explicit renaming relations occur in 261 sentences, synonymy-like relations occur in 349 sentences, and biological proof-based relations occur in 76 sentences.

**Table 3:** Positive examples of the Rename task.

***Explicit renaming***
*PMID 15767583*: Genetic analysis of ykvJKLM mutants in Acinetobacter confirmed that each was essential for queuosine biosynthesis, and the genes were renamed *queCDEF*.
***Implicit renaming***
*PMID 8002615*: Analysis of a suppressor mutation ssb (*kinC*) of *sur0B20 *(spo0A) mutation in *Bacillus subtilis *reveals that kinC encodes a histidine protein kinase.
***Biological proof***
*PMID 1744050*: DNA sequencing established that spoIIIF and *spoVB *are a single monocistronic locus encoding a 518-amino-acid polypeptide with features of an integral membrane protein.

The underlined names are the former names, and the emphasized names are the new names.

**Explicit renaming **relation is the easiest positive case to identify. In the example, the aggregation of gene names "ykvJKLM" is clearly renamed by the authors as "queCDEF". Although the four genes are concatenated, there is no evidence mentioned of them acting as an operon. Furthermore, despite the context involving mutants of *Acinetobacter*, the aggregation belongs correctly to *B. subtilis*.

**Implicit renaming **is an asymmetric relation because one of the synonyms is intended to replace the other synonym in future uses. The example presents two renaming relations between former names "ssb" and "spo0A", and new names "kinC" and "sur0B20", respectively. The renaming relation between "ssb" and "kinC" has a different orientation because of additional information in the reference. Similar to the preceding example, the renaming is a consequence of a genetic mutation experiment. The description of mutation experiments represent an important source of difficult annotations.

**Biological proof **is a renaming relation induced by an explicit scientific conclusion while the renaming is not, as in the example where experiments reveal that two loci "spoIIIF" and "spoVB" are in fact the same locus, and they become synonyms. Terms such as "allelic to" or "identical to" usually qualify such conclusions. Predicting biological proof-based relations requires some biological modeling.

Finally, mutations are frequent in Microbiology for revealing gene phenotypes. They carry information about the original gene names (e.g., "rvtA11" is a mutant name created by adding "11" to "rvtA"). However, partial names cannot be partially annotated, in other words, the original part ("rvtA") would not be annotated in the mutation name ("rvtA11"). Most of these names are local names, and should not be annotated because of their restricted scope. It could happen that the mutation name is registered as a synonym in several international databases. To avoid inconsistencies, all of the renamings involving a mutation referenced in a database were accepted, and only biological proof-based and explicit renamings involving a strict non-null unreferenced mutation (a null mutation corresponds to a total suppression of a gene) were accepted.

To simplify the task, only short names of gene/protein/groups in *B. subtilis *were considered. Naming conventions set up short names of four letters along with an upper case letter at the end for all of the genes (*e.g*., "gerE"), and the same names with the upper case of the initial letter (*e.g*., "GerE") and long names for the proteins (*e.g*., "Spore germination protein GerE"). However, many irregular gene names exist (*e.g*., "tuf", "spoIIAC"), which are considered as well. It also happens that gene or protein name lists are abbreviated by factorizations to form a sequence. For example, "queCDEF" is the abbreviation of the list of gene names "queC", "queD", "queE" and "queF". Such aggregations are acceptable gene names as well. In any case, these details were not needed by the task participants because the corpus was provided with tagged gene names. Most renaming relations involve pairs of the same type, genes, proteins or aggregations, except for 18 cases.

Multiple occurrences of the same renaming relation were annotated independently and had to be predicted. The renaming pairs are directed, and the former and the new forms must be distinguished. When the renaming order was not explicit in the document, the rule was to annotate by default the first member of the pair as the new form and the second member as the former form.

#### Prediction evaluation metrics

The evaluation of the Rename task is given in terms of the recall, precision and F-score of the renaming relations. Two set of scores are given: the first set is computed by enforcing a strict direction of the renaming relations, and the second set is computed with a relaxed direction. Because the relaxed score accounts for renaming relations even if the arguments are inverted, it will necessarily be greater than or equal to the strict score. The participant's score is the relaxed score, and the strict score is given for information. Relaxed scores are informative with respect to the application goal. The choice of the canonical name, among the synonyms, for denoting a gene is conducted by the bacteriology community, and it can be independent of the anteriority or novelty of the name. The annotation of the reference corpus showed that the direction was not always decidable, even for a human reader. Thus, it would have been unfair to evaluate systems on the basis of uncertain information.

### Bacteria Gene Interaction

The corpus roughly contains three types of genic interaction mentions, namely regulations, regulon membership and binding. The first case corresponds to interactions that have a mechanism that is not explicitly given in the text. The mention of the genic interaction in this case only tells that the transcription of a given gene is influenced by a given protein, either positively (activation), negatively (inhibition) or in an unspecified way. The second type of genic interaction mentioned (regulon membership) basically conveys the same information, using the regulon term/concept. The regulon of a gene is the set of genes that it controls. In that case, the interaction is expressed by saying that a gene is a member of some regulon. The third and last type of genic interaction mentioned provides more mechanistic details on a regulation because it describes the binding of a protein near the promoter of a target gene. This structure motivates the introduction of *Promoter *and *Site *entities, which correspond to DNA regions. It is, thus, possible to extract the architecture of a regulatory DNA region, linking a protein agent to its gene target (see Figure 2).

The set of entity types is divided into two main groups, namely 10 genic entities and 3 types of action (Table 4). Genic entities represent biological objects such as a gene, a group of genes or a gene product. Specifically, a *GeneComplex *annotation corresponds to an operon, which is a group of genes that are contiguous in the genome and under the control of the same promoter. The annotation *GeneFamily *is used to denote either genes involved in the same biological function or genes with sequence homologies. More importantly, *PolymeraseComplex *annotations correspond to the protein complex that is responsible for the transcription of genes. This complex includes several subunits (components), which are combined with a sigma factor; that recognizes specific promoters on the DNA sequence.

**Table 4:** List of molecular entities and actions in the Gene Interaction corpus.

*Name*	*Example*
*Gene*	cotA
*GeneComplex*	sigX-ypuN
*GeneFamily*	class III heat shock genes
*GeneProduct*	yvyD gene product
*Protein*	CotA
*PolymeraseComplex*	SigK RNA polymerase
*ProteinFamily*	DNA-binding protein
*Site*	upstream site
*Promoter*	promoter regions
*Regulon*	regulon

*Action*	activity | level | presence
*Expression*	expression
*Transcription*	transcription

The second group of entities are phrases that express either molecular processes (e.g. sequestration, dephosphorylation) or the molecular state of the bacteria (*e.g*., the presence, activity or level of a protein). They represent some type of action that can be performed on a genic entity. Note that transcription and expression events were tagged as specific actions because they play a specific part in certain relations (see below).

The annotation of entities and actions was provided to the participants, and the task consisted of extracting the relations listed in Table 5.

**Table 5:** List of relations in the Gene Interaction corpus.

*Name*	*Example*
*ActionTarget*	**expression **of *yvyD*
*Interaction*	**ComK **negatively regulates *degR *expression
*RegulonDependence*	*sigmaB ***regulon**
*RegulonMember*	*yvyD *is member of sigmaB **regulon**
*BindTo*	**GerE **adheres to the *promoter*
*SiteOf*	**-35 sequence **of the *promoter*
*PromoterOf*	the *araE ***promoter**
*PromoterDependence*	*GerE*-controlled **promoter**
*TranscriptionFrom*	**transcription **from the *upstream site*
*TranscriptionBy*	**transcription **of cotD by *sigmaK RNA polymerase*

The relations are binary and directed and rely on the entities defined above. The three types of interactions are represented with an *Interaction *annotation, linking an agent to its target. The other relations provide additional details on the regulation, such as elementary components involved in the reaction (sites, promoters) and contextual information (mainly provided by the *ActionTarget *relations). A formal definition of relations and relation argument types can be found on the BioNLP 2011 Shared Task Web page http://sites.google.com/site/bionlpst.

#### Corpus annotation methodology

The source of the GI corpus is a set of PubMed abstracts that address the transcription of genes in *Bacillus subtilis*. The semantic annotation, derived from the ontology of [8], contains 10 molecular entities, 3 different actions, and 10 specialized relations. This annotation is applied to 162 sentences from the LLL set [1], which are provided with manually checked linguistic annotations (segmentation, lemmatization, syntactic dependencies). The corpus was split into 105 sentences for training, 15 for development and 42 for test. Table 6 gives the distribution of the entities and actions per corpus, and Table 7 gives the distribution of the relations per corpus.

**Table 6:** Distribution of entities and actions in the Gene Interaction corpus.

*Entity or action*	***Train. + Dev***.	*Test*
Documents	(105+15) 120	42
*Protein*	219	85
*Gene*	173	56
*Transcription*	53	21
*Promoter*	49	10
*Action*	45	22
*PolymeraseComplex*	43	14
*Expression*	29	6
*Site*	22	8
*GeneComplex*	19	4
*ProteinFamily*	12	3
*Regulon*	11	2
*GeneProduct*	10	3
*GeneFamily*	6	5

**Table 7:** Distribution of the relations in the Gene Interaction corpus.

*Relation*	***Train. + Dev***.	*Test*
*Interaction*	208	64
*ActionTarget*	173	47
*PromoterOf*	44	8
*BindTo*	39	4
*PromoterDependence*	36	4
*TranscriptionBy*	36	8
*SiteOf*	23	6
*RegulonMember*	17	2
*TranscriptionFrom*	14	2
*RegulonDependence*	12	1

The semantic annotation scheme was developed by two annotators through a series of independent annotations of the corpus, followed by reconciliation steps, which could involve concerted modifications [6]. As a third and final stage, the corpus was reviewed and the annotation was simplified to make it more appropriate to the contest. The final annotation contains 748 relations distributed in nine categories, 146 of them belonging to the test set.

The annotation scheme was well suited to accurately represent the meaning of the sentences in the corpus, with one notable exception. In the corpus, there is a common phrasing to express that a protein P regulates the transcription of a gene G by a given sigma factor S. In that case, the only annotated interactions are between the pairs (P, G) and (S, G). This representation is not completely satisfactory, and a ternary relation involving P, S and G would have been more adequate.

Additional specific rules were needed to cope with linguistic issues. First, when the argument of a relation had coreferences, the relation was repeated for each maximally precise coreference of the argument. Second, in case of a conjunction such as "sigmaA and sigmaX holoenzymes", there should ideally be two entities (namely "sigmaA holoenzyme" and "sigmaX holoenzyme"); however, this scenario is not easy to represent using the BioNLP format. In this situation, we grouped the two entities together. These cases were rare and were unlikely to affect the feasibility of the task because entities were provided in the test set.

#### Prediction evaluation metrics

The training and development corpora with the reference annotations were made available to participants by December, 1st on the BioNLP shared Task pages together with evaluation software. The test corpus with the entity annotations has been made available by March 1st. The participants sent the predicted annotations to the BioNLP shared Task organizers by March 10th. The evaluation results were computed and were provided to the participants and on the Web site the same day. The participants are evaluated and ranked according to two scores: the F-score for all of the event types together and the F-score for the *Interaction *event type. For a predicted event to count as a hit, both arguments must be the same as in the reference and in the right order, and the event type must be the same as in the reference.

### Bacteria Biotopes

#### Corpus annotation methodology

**Figure 4:**
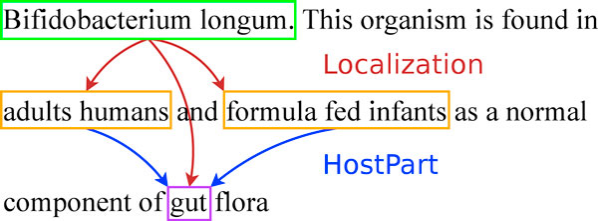
**Example of bacteria biotopes relations**.

**Figure 5:**
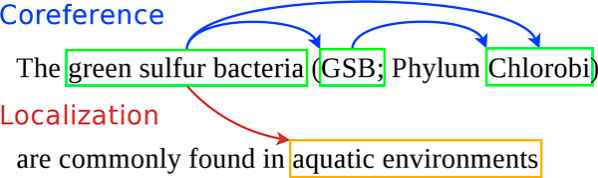
**Example of a coreference**.

The goal of the BB task is illustrated in Figure 4. The entities to be extracted are of two main types: bacteria and locations. They are text-bound, and their position must be predicted. Relations are of type *Localization *between bacteria and locations, and *PartOf *between hosts and host parts. In the example in Figure 4, *Bifidobacterium longum *is a bacterium, and *adult humans *and *formula fed infants *denote host locations for the bacteria. Additionally, *gut *is a bacteria location, is part of the two hosts and thus is of type *HostPart*. Coreference relations between entities denoting the same information represent valid alternatives for the relation arguments. For example, the three taxon name occurrences in Figure 5 are equivalent because they actually designate the same taxon. The coreferences are provided in the training and development sets, but they do not have to be predicted in the test set.

The training, development and test documents are Web pages of bacteria sequencing projects:

• Genome Projects referenced at NCBI http://www.ncbi.nlm.nih.gov/genomes/lproks.cgi;

• Microbial Genomics Program at JGI http://genome.jgi-psf.org/programs/bacteria-archaea/index.jsf;

• Bacteria Genomes at EBI http://bacteria.ensembl.org/index.html;

• Microorganisms sequenced at Genoscope http://www.genoscope.cns.fr/spip/Microorganisms-sequenced-at.html;

• Encyclopedia pages from MicrobeWiki http://microbewiki.kenyon.edu/index.php/MicrobeWiki.

The documents are publicly available and quite easy to understand by non-experts compared to scientific papers on similar topics. From the 2,086 downloaded documents, 105 were randomly selected for the BB task. A quarter of the corpus was retained for test evaluation. The remainder was split into training and development sets.

The BB task requires the locations to be assigned a type among eight types that capture high-level information for further ontology mappings. The location types are *Host, HostPart, Geographical *and *Environment*. *Environment *is broadly defined to qualify locations that are not associated with hosts, in a similar way as described in [13]. The *Environment *class is divided into *Food, Medical, Soil *and *Water*. Locations that are none of these subtypes are classified as *Environment*.

The important information conveyed by the locations, especially of the *Environment *type, is the function of the bacterium in its ecosystem rather than the substance of the habitat. Indeed, the final goal is to extract habitat properties and bacteria phenotypes. Beyond the identification of locations, their properties (*e.g*., temperature, pH, salinity, oxygen) are of high interest for phenotypes (*e.g*., thermophily, acidophily, halophily) and trophism studies. This information is difficult to extract and is often incomplete or even not available in papers [10]. Hopefully, some properties can be automatically retrieved with the help of specialized databases, which give the physico-chemical properties of locations, such as hosts (plant, animal, human organs), soils (see WebSoilSurvey: http://websoilsurvey.nrcs.usda.gov/, Corine Land Cover: http://www.eea.europa.eu/data-and-maps/data#c12=corine+land+cover+version+13), water, or chemical pollutants.

HTML tags and irrelevant metadata were stripped from the corpus. The Alvis pipeline [14] pre-annotated the species names that are potential bacteria and host names. A team of 7 scientists manually annotated the entities, coreferences and relations using the Cadixe XML editor. Each document was processed by two independent annotators in a double-blind manner. Conflicts were automatically detected and resolved by annotator negotiation and irrelevant documents (*e.g*., without a bacterial location) were removed. The remaining inconsistencies among the documents were resolved by the two annotators, who were assisted by a third person acting as an arbitrator.

The annotator group designed the detailed annotation guidelines in two phases. First, they annotated a set of 10 documents, discussed the options and wrote detailed guidelines with representative and illustrative examples. During the annotation of the remainder of the documents, new cases were discussed by email, and the guidelines were amended accordingly.

##### Location types

The main issues under debate were the definitions of the location types, the boundaries of annotations and coreferences. Additional annotation specifications concerned the exclusion of overly general locations (*e.g*., *environment, zone*), artificially constructed biotopes and indirect effects of bacteria on distant places. For example, a disease symptom occurring in a given host part does not imply the presence of the bacteria in this place, whereas an infection does. The boundaries of the types were also an important point of discussion because the definite formalization of the habitat categories was at stake. For example, we decided to exclude land environment citations (*e.g*., *fields, deserts, savannah*) from the type *Soil *, and thus, we enforced a strict definition of soil bacteria. The most controversial type was the host parts. We decided to include fluids, secretions and excretions (which are not strictly organs). Therefore, the host parts category required specifications to determine at which point of dissociation from the original host a habitat is not longer a host part (*e.g*., *mother's milk *vs. *industrial milk, rhizosphere *as *HostPart *instead of *Soil*).

##### Boundaries

The bacteria name boundaries do not include any external modifiers (*e.g*., *two A. baumannii strains*). Irrelevant modifiers of locations are considered to be outside the annotation boundaries (*e.g*., *responsible for a hospital epidemic*). All of the annotations are contiguous and span a single fragment in the same way as the other BioNLP Shared Tasks. This constraint led us to consider cases in which several annotations occur side by side. The preferred approach was to have one distinct annotation for each different location (*e.g*., *contact with infected animal products or through the air*). In the case of head or modifier factorization, the annotation depends on the information conveyed by the factorized part. If the head is not relevant to determine the location type, then each term is annotated separately (*e.g*., *tropical and temperate zones*). Conversely, if the head is the most informative with regard to the location type, a single annotation spans the whole fragment (*e.g*., *fresh and salt water*).

##### Coreferences

Two expressions are considered to be coreferential and thus valid solution alternatives if they convey the same information. For example, complete taxon names and non-ambiguous abbreviations are valid alternatives (*e.g*., *Borrelia garinii *vs. *B*. *garinii*), while ambiguous anaphora ellipses are not (*e.g*., as in "... *infected with Borrelia duttonii*. *Borrelia then multiplies*..."). The ellipsis of the omitted specific name (*dutotonii*) leaves the ambiguous generic name (*Borrelia*).

The full guidelines document is available for download on the BioNLP Shared Task Bacteria Biotope page http://sites.google.com/site/bionlpst/home/bacteria-biotopes/BioNLP-ST_2011_Bacteria_Biotopes_Guidelines.pdf.

#### Annotated corpus analysis and annotator agreement

Table 8 gives the distribution of the entities and relations per corpus. The distribution of the five document sources in the test corpus reflects the distribution of the training set and no other criteria. *Food *is therefore underrepresented.

**Table 8:** Bacteria Biotope corpus size.

	*Train + Dev*	*Test*
Documents	78 (65 + 13)	27 (26%)

Bacteria	538	121 (18%)

Environment	62	16 (21%)
Host	486	101 (17%)
HostPart	217	84 (28%)
Geographical	111	25 (18%)
Water	70	21 (23%)
Food	46	0 (0%)
Medical	24	2 (8%)
Soil	26	20 (43%)

Coreferences	484	100 (17%)
Total entities	1,580	390

Localization	998	250 (20%)
Part of Host	204	78 (28%)
Total relations	1,202	328

Because differences could be in the location types, entity boundaries, event arguments and coreference chains, typical inter annotator agreement (IAA) scores such as alpha and kappa would have been meaningless. Indeed, these measure the agreement between the annotators that classify items; however, the annotation in the BB corpus assigned boundaries, categories, specific relations and equivalence relations (coreferences). Instead, we used the evaluation metrics described in the next section to score the annotation of one annotator, using the concurrent annotations as reference. Table 9 outines the obtained scores. The overall score of 74% is unexpectingly high with regard to the complexity of the annotation task and the many points where annotators could be in disagreement. *Environment *and *Food *location types stand out for the lowest agreement scores. The *Environment *type suffered because of its status as a "default" type: annotators tended to annotate as *Environment *locations for which they had a doubt about the type. For *Food*, the differences are very diverse (typing, boundaries and bacteria attribution); the only notably recurrent difference was the frequent confusion between *Food *and *Water *(as in "bottle water") or *HostPart *(as in "milk"). Finally, we note that the *Bacterium *entity recall is at the same level as the best team (see Table 10).

**Table 9:** Inter Annotator Agreement for the Bacteria Biotopes corpus.

	*Entity recall*	*Event recall*	*Event precision*	*F-score*
*Bacterium*	81			

*Host*	88	76	77	77
*HostPart*	85	78	83	80
*Geographical*	88	73	75	74
*Environment*	54	51	52	52
*Food*	69	46	52	49
*Medical*	87	74	80	77
*Water*	83	75	67	71
*Soil*	83	71	70	71

*PartOf*		72	76	74
*Global*		73	75	74

**Table 10:** Bacteria entity recall of the participants of the Bacteria Biotope task.

Bibliome	84
JAIST	55
UTurku	16

#### Prediction evaluation metrics

Participants were provided the training and development sets annotated with entities, events and coreferences. The test set was provided as raw text. The evaluation metrics are based on precision, recall and the F-measure. Predicted entities that are not event arguments are ignored and they do not penalize the score. Each event *E_r _*in the reference set is matched to the predicted event *E_p _*that maximizes the event similarity function *S*. The recall is the sum of the *S *results divided by the number of events in the reference set. Each event *E_p _*in the predicted set is matched to the reference event *E_r _*that maximizes *S*. The precision is the sum of the *S *results divided by the number of events in the predicted set. Participants were ranked by the F-score, defined as the harmonic mean between the precision and recall. *E_ab_*, the event similarity function between a reference *Localization *event *a *and a predicted *Localization *event *b*, is defined as follows:

$$E_{ab}=B_{ab}.T_{ab}.J_{ab}$$

*B_ab _*is the bacteria boundary component defined as follows: if the Bacterium arguments of both the predicted and reference events have exactly the same boundaries, then *B_ab _*= 1; otherwise, *B_ab _*= 0. Bacteria name boundary-matching is strict because boundary mistakes usually yield a different taxon. *T_ab _*is the location type prediction component, which is defined as follows: if the Location arguments of both the predicted and reference events are of the same type, then *T_ab _*= 1; otherwise, *T_ab _*= 0.5. Thus, type errors divide the score by two.

*J_ab _*is the location boundary component defined as follows: if the Location arguments of the predicted and reference events overlap, then we have the following:

$$J_{ab}=\frac{OV_{ab}}{LEN_{a}+LEN_{b}-OV_{ab}}$$

where *LEN_a _*and *LEN_b _*are the length of the *Localization *arguments of the predicted and reference events and *OV_ab _*is the length of the overlapping segment between the *Localization *arguments of the predicted and reference events. If the arguments do not overlap, then *J_ab _*is 0. This formula is a Jaccard index applied to overlapping segments. Location boundary matching is relaxed, although it rewards predictions that approach the reference.

For the *PartOf *events between *Hosts *and *HostParts*, the event similarity function *P_ab _*is defined as follows: if the *Host *arguments of the reference and predicted events overlap and the *Part *arguments of the reference and predicted events overlap, then *P_ab _*= 1; otherwise, *P_ab _*= 0. The boundary matching of the *PartOf *arguments is relaxed because boundary mistakes are already penalized in *E_ab_*.

Arguments that belong to the same coreference set are strictly equivalent. In other words, the argument in the predicted event is correct if it is equal to the reference entity or to any item in the reference entity coreference set.

## Results

### Bacteria Gene Renaming

Final submissions were received from three teams, the University of Turku (Uturku), the University of Concordia (Concordia) and the Bibliome team from MIG/INRA. Their results are summarized in Table 11. The ranking order is given by the overall F-score for relations with a relaxed argument order. Uturku achieved the best F-score with a very high precision and a high recall. Concordia achieved the second F-score with balanced precisions and recalls. Bibliome is five points behind with a better recall but much lower precision. Both UTurku and Concordia predictions rely on dependencies (Charniak-Johnson and Stanford, respectively, using the McClosky model), whereas Bibliome predictions rely on a bag of words. This demonstrates the high value of dependency-parsing for this task, especially for the precision of predictions. We notice that the UTurku system uses machine learning (SVM) and Concordia uses rules based on trigger words. The good results of UTurku confirms the hypothesis that gene renaming citations are highly regular in the scientific literature. The most frequently missed renamings belong to the Biological Proof category (see Table 3). This result is expected because the renaming is formulated as a reasoning whereas the conclusion is only implicit.

**Table 11:** Participant scores at the Rename task.

*Team*	***Prec***.	*Recall*	*F-score*
Univ. of Turku	**95.9**	**79.6**	**87.0**
Concordia Univ.	74.4	65.9	69.9
INRA	57.0	73.9	64.4

The very high score of the Uturku method leads us to conclude that the task can be considered as solved by a linguistic-based approach. Whereas Bibliome used an extensive nomenclature considered to be exhaustive and sentence filtering using a SVM, Uturku used only two nomenclatures in synergy but with more sophisticated linguistic-based methods, in particular syntactic analyses. Bibliome methods showed that a too high dependence to nomenclatures can decrease scores if they contain compromised data. However, the use of an extensive nomenclature as done by Bibliome may complement the Uturku approach and improve recall. It is also interesting that both systems do not manage renamings that cross sentence boundaries. The good results of the renaming task will be exploited to keep synonym gene lists up to date with extensive bibliography mining. More Specifically, this approach will contribute to enriching SubtiWiki, a collaborative annotation effort on *B. subtilis *[15,16].

### Bacteria Gene Interaction

There was only one participant in the bacterial gene interaction, whose results are shown in Tables 12 and 13. Some relations were not significantly represented in the test set, and thus, the corresponding results should be considered with caution. This scenario is the case for *RegulonMember *and *TranscriptionFrom*, which are only represented two times each in the test. The lowest recall, 17%, obtained for the *SiteOf *relation, is explained by its low representation in the corpus: most of the test errors come from a difficult sentence with coreferences.

**Table 12:** University of Turku global scores at the Gene Interaction task.

*Event*	*U. Turku scores*
Global Precision	85
Global Recall	71
Global F-score	77

Interaction Precision	75
Interaction Recall	56
Interaction F-score	64

**Table 13:** University of Turku detailed scores at the Gene Interaction task.

*Event*	***Prec***.	***Rec***.	*F-score*
Global	85	71	77
*ActionTarget*	94	92	93
*BindTo*	75	75	75
*Interaction*	75	56	64
*PromoterDependence*	100	100	100
*PromoterOf*	100	100	100
*RegulonDependence*	100	100	100
*RegulonMember*	100	50	67
*SiteOf*	100	17	29
*TranscriptionBy*	67	50	57
*TranscriptionFrom*	100	100	100

**Figure 6:**
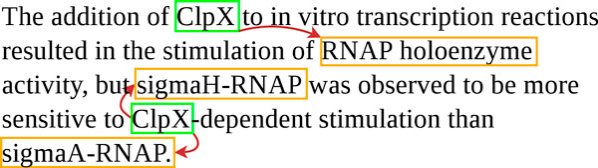
**Examples of commonly missed gene interactions**.

The recall of 56% for the *Interaction *relation certainly illustrates the heterogeneity of this category, which gathers mentions of interactions at large as well as precise descriptions of gene regulations. For example, Figure 6 shows a complex instance in which all of the interactions were missed. Surprisingly, we also found false negatives in rather trivial examples ("*ykuD was transcribed by SigK RNA polymerase from T4 of sporulation*."). Uturku used an SVM-based approach for extraction, and it is, thus, difficult to account for the false negatives in a simple and concise way.

The GI corpus was previously used in a relation extraction work [7] based on Inductive Logic Programming [17]. However, a direct comparison of the results is not appropriate here because the annotations were partially revised, and the evaluation setting was different (leave-one-out in Manine's work, test set in the challenge).

Nevertheless, we note similar tendencies if we compare relative results between relations. Specifically, it was also found in Manine's paper that *SiteOf, TranscriptionBy *and *Interaction *are the most difficult relations to extract. It is also worth mentioning that both approaches rely on syntactic dependencies and use the curated dependencies provided in the corpus. Interestingly, the approach by the University of Turku reports a slightly lower F-measure with dependencies calculated by the Charniak parser (approximately 1%, personal communication). This information is especially important when considering a production setting. The quality of the results for both of the challenges suggests that current methods are mature enough to be used in semi-automatic strategies for genome annotation, where they could efficiently assist biological experts involved in collaborative annotation efforts [16]. However, the false positive rate, notably for the *Interaction *relation, is still too high for the extraction results to be used as a reliable source of information without a curation step.

### Bacteria Biotopes

Three teams submitted predictions to the BB task. The first team is from the University of Turku (UTurku); their system is generic and produced predictions for every BioNLP Shared Task. This system intensely uses Machine Learning, especially SVMs, for entity recognition, entity typing and event extraction. UTurku adapted their system for the BB task by using specific NER patterns and external resources [18].

The second team is from the Japan Advanced Institute of Science and Technology (JAIST); their system was Specifically designed for this task. They used CRFs for entity recognition and typing, and classifiers for coreference resolution and event extraction [19].

The third team is from Bibliome INRA; their system was Specifically designed for this task [20]. This team has the same affiliation as the BB task authors; however, great care was taken to prevent communication on the subject between task participants and the test set annotators.

The results of the three submissions according to the official metrics are shown in Table 14. The scores are micro-averaged: *Localization *and *PartOf *relations have the same weight. The Bibliome team achieved the highest F-measure with a balanced recall and precision (45%). Given the novelty and the complexity of the task, these first results are quite encouraging. Almost half of the relations are correctly predicted.

**Table 14:** Results of the participants of the Bacteria Biotope task.

	*Recall*	*Precision*	*F-score*
Bibliome	**45**	45	**45**
JAIST	27	42	33
UTurku	17	**52**	26

All three systems perform the same distinct sub-tasks: bacteria name detection, detection and typing of locations, coreference resolution and event extraction. The following description of the approaches used by the three systems in each subtask will be supported by intermediate results.

#### Bacteria name detection

Interestingly, the three participants used three different resources for the detection of bacteria names: the List of Prokaryotic Names with Standing in Nomenclature (LPNSN) by UTurku, names in the genomic BLAST page of NCBI by JAIST and the NCBI Taxonomy by Bibliome.

Table 10 shows a disparity in the bacteria entity recall of participants. The merits of each resource cannot be deduced directly from these figures because they have been exploited in different ways. UTurku and JAIST systems injected the resource as features in a ML algorithm, whereas Bibliome directly projected the resource on the corpus with additional patterns to detect abbreviations.

However, there is some evidence that the resources have a major impact on the result. According to [21], LPNSN is necessarily incomplete. NCBI BLAST contains only names of species for which a complete genome has been published. The NCBI Taxonomy used by INRA contains only names of taxa for which a sequence was published. It appears that all of the lists are incomplete. However, the bacteria referenced by the sequencing projects, which are mentioned in the corpus, should all be recorded by the NCBI Taxonomy.

#### Location detection and typing

As stated before, locations are not necessarily denoted by strict named entities. This consideration was an interesting challenge that called for the use of external resources and linguistic analysis with a broad scope.

UTurku and JAIST both used WordNet, a sensible choice because it encompasses a wide vocabulary and is also structured with synsets and hyperonymy relations. The WordNet entries were injected as features in the participant ML-based entity recognition and typing subsystems.

It is worth noting that JAIST also used word clustering based on Maximum Entropy Markov Models for entity detection, which can be viewed as a form of distributional semantics. JAIST experiments demonstrated a slight improvement using word clustering, and further exploration of this idea could prove to be valuable.

Alternatively, the Bibliome system used a combination of linguistic criteria, such as term compositionality and reasoning over an ontology, to predict the locations boundaries and types. Bibliome also used additional resources for specific types: the NCBI Taxonomy for type *Host*, Agrovoc countries for type *Geographical *and a custom ontology for all of the other types.

The location entity recall in Table 15 shows that Bibliome consistently outperformed the other groups for all of the types except for *Geographical*. This result demonstrates the strength of exploiting a resource with strong semantics (ontology vs. lexicon) and with mixed semantic and linguistic rules.

**Table 15:** Location entity recall of the participants of the Bacteria Biotope task.

	*Bibliome*	*JAIST*	*UTurku*
Host	82	49	28
Host part	72	36	28
Geographical	29	60	53
Environment	53	10	11
Water	83	32	2
Soil	86	37	34

To evaluate the impact of the location entity boundaries and types, we computed the final score by relaxing the *T_ab _*and *J_ab _*measures. We re-defined *T_ab _*as being always equal to 1, in other words, the type of the localization was not evaluated. We also re-defined *J_ab _*as the following: if the *Localization *arguments overlap, then *J_ab _*= 1; otherwise, *J_ab _*= 0. This definition means that the boundaries were totally relaxed. The relaxed scores are shown in Table 16. While the difference is not significant for JAIST and UTurku, the Bibliome results exhibit a 9 point increase. This increase demonstrates that the Bibliome system is efficient at predicting which entities are locations, while the other participants predict more accurately the boundaries and types.

**Table 16:** Relaxed scores of the participants of the Bacteria Biotope task.

	*Recall*	*Precision*	*F-score*	*Difference*
Bibliome	54	54	54	+9
JAIST	29	45	35	+2
UTurku	19	56	28	+2

#### Coreference resolution

The corpus exhibits an unusual number of anaphora, especially bacteria coreferences because a single bacterium species is usually the central topic of a document. The Bibliome submission is the only system that performs bacteria coreference resolution. Their system is rule-based and addresses the with referential "it", bi-antecedent anaphora and sortal anaphora. The JAIST system has a bacteria coreference module based on ML; however, its submission was performed without coreference resolution because their experiments did not show any improvement.

#### Event extraction

Both UTurku and JAIST approached the event extraction as a classification task using ML (SVM). Bibliome exploited the co-occurrence of arguments and the presence of trigger words from a predefined list. Both UTurku and Bibliome generate events in the scope of a sentence, whereas JAIST generates events in the scope of a paragraph.

As shown in Table 17, UTurku achieved the best score for the *PartOf *events. For all of the participants, the prediction is often correct (between 60 and 80%) while the recall is rather low (20 to 32%). Conversely, the score of the *Localization *relation by UTurku has been penalized by its low recognition of bacteria names (16%). This shortcoming strongly affects the score of *Localizations *because one of the arguments is necessarily a bacterium. The good results of Bibliome are partly explained by its high bacteria name recall of 84%.

**Table 17:** Detailed scores of the participants of the Bacteria Biotope task.

	*Bibliome*			*JAIST*			*UTurku*		
	*Recall*	*Precision*	*F-score*	*Recall*	*Precision*	*F-score*	*Recall*	*Precision*	*F-score*
Host	**61**	48	**53**	30	43	36	15	**51**	23
HostPart	**53**	42	**47**	18	**68**	28	9	40	15
Geographical	13	38	19	**52**	35	**42**	32	**40**	36
Environment	**29**	24	**26**	5	0	0	6	**50**	11
Water	**60**	**55**	**57**	19	27	23	1	7	2
Soil	**69**	**59**	**63**	21	42	28	12	21	15
PartOf	23	79	36	31	61	41	**32**	**83**	**46**

The lack of coreference resolution could penalize the event extraction recall. To test this hypothesis, we computed the recall by accounting for only the events in which both arguments occur in the same sentence. The goal of this selection is to remove most of the events that are denoted through a coreference. The recall difference was not significant for Bibliome and JAIST; however, UTurku recall was raised by 12 points (29%). That experiment confirms that UTurku's low recall is explained by coreferences rather than the quality of its event extraction. The paragraph scope chosen by JAIST most likely compensates for the lack of coreference resolution.

As opposed to Bibliome, the precision of the *Localization *relation prediction by JAIST and UTurku; is high compared to the recall, with a noticeable exception in the geographical locations. The difference between the participants appears to be caused by the geographical entity recognition step more than by the relation itself. This conclusion is shown by the difference between the entity and the event recall (Tables 15 and 17, respectively). The worst predicted type is *Environmental *, which includes diverse locations, such as agricultural, natural and industrial sites and residues. This result reveals significant room for improvement for *Water, Soil *and *Environmental *entity recognition.

## Conclusions

In this paper, we described the BioNLP 2011 Shared Task Bacteria Track, the first Information Extraction challenge that is entirely dedicated to bacteria. The track consists of three tasks: Gene Renaming, Gene Interaction, and Biotopes, which are of increasing richness:

• in the Gene Renaming task, there is a single relation to extract, and each *Renaming *relation occurs within a single sentence; this relation is expressed in relatively standard ways; also, the arguments are single, normalized words, which are provided to the participants.

• while the arguments are also given in the Gene Interaction task, there are 10 specialized relations to extract, some of which could be expressed in very diverse ways (*e.g*., *SiteOf*).

• in the Biotope task, finding arguments is an especially serious added difficulty because it involves addressing linguistic challenges such as terminological analysis and coreference resolution between sentences (in contrast to the other tasks in which coreferences are always bound to the sentence).

Unsurprisingly, the results of participating systems showed that the tasks are also increasingly difficult to tackle: while the task can be considered as solved for Gene Renaming, the results concerning Biotope still leave a large amount of room for improvement.

This progression reflects a desirable goal in biological information extraction. As a first aspect, defining tasks using an annotation schema with specialized relations contributes to improved capturing of the implicit representations used by the authors of the publications, thereby improving the information extraction efficiency. While formalizing concepts in an explicit schema could be difficult, the annotation process is not necessarily more complex, once the guidelines are established.

A second aspect in this track is the shift from single-word, normalized arguments such as gene identifiers to biological entities that can appear under many different forms. Retrieving the latter type of argument requires an advanced terminological analysis as well as appropriate normalization procedures, for example, for associating the terms extracted from the text with concepts in an ontology. In the Biotope task, the participants were asked to find localization terms and to categorize them using a simple hierarchy of microorganism habitats, including eight location concepts. The same approach could be considered for gene interactions, allowing us to introduce non-genic actors such as environmental factors and stresses, molecules, biological functions, metabolic pathways, or cellular structures. The major benefit of accounting for such actors would be to integrate the molecular regulation networks into their biological and cellular context.

In our opinion, the current available technologies for extraction are becoming mature enough to consider applications in a production setting, beyond challenges on controlled corpora. In this context, a wealth of extraction tasks could be needed, encompassing various difficulty levels. As an example, the corpus of the Gene Renaming task mentions only the genes of *B. subtilis*, while a production system should cope with homonymous gene names belonging to distinct species, using disambiguation procedures. On another level, the Biotope task could be extended by considering the extraction of physico-chemical properties of the habitats and, conversely, the characterics of the bacteria they induce, in terms of metabolism and trophisms.

Extraction systems that are based on fine-grained schema and that address non-standardized formulations of concepts could lead to several applications with high practical impact. These applications include semantic search engines tailored for specific scientific domains or the development of normalization procedures for semi-structured information in databases.

## Competing interests

The authors declare that they have no competing interests.

## Authors' contributions

PB, RB, JJ, CN and PV have written this document.

RB has supervised the annotation of the Bacteria Biotope corpus. RB, MvdG, JJ and CN have designed the annotation guidelines and have annotated the Bacteria Biotopes corpus.

JJ and CN have supervised the annotation of the Bacteria Rename corpus. JJ has validated the annotations of the Bacteria Rename corpus.

PB and PV have supervised the annotation of the Bacteria Gene Interaction corpus. EA, PB, RB, APM and PV have annotated the Bacteria Gene Interaction corpus.

RB has supported the infrastructure -corpus release and evaluation, for the Bacteria Track tasks within the BioNLP-ST 2011.
